# High NK cell counts at day 90 predict improved survival in event-free patients after T-cell depleted allogeneic stem cell transplantation

**DOI:** 10.3389/fimmu.2025.1577924

**Published:** 2025-06-18

**Authors:** Andrej Pešić, Nevena Bešević, Nicolaus Kröger, Dejana Stanisavljević, Nada Kraguljac Kurtović, Zoran Bukumirić, Natalija Kecman, Nikola Lemajić, Mihailo Smiljanić, Nada Suvajdžić Vuković, Andrija Bogdanović, Ana Vidović, Jelena Bila, Mirjana Mitrović, Danijela Leković, Marijana Virijević, Irena Đunić, Darko Antić, Milena Todorović Balint

**Affiliations:** ^1^ Clinic for Hematology, University Clinical Center of Serbia, Belgrade, Serbia; ^2^ Department of Stem Cell Transplantation, University Medical Center Hamburg-Eppendorf, Hamburg, Germany; ^3^ Faculty of Medicine, University of Belgrade, Belgrade, Serbia; ^4^ Institute for Medical Statistics and Informatics, Faculty of Medicine, University of Belgrade, Belgrade, Serbia

**Keywords:** NK cells, allogeneic stem cell transplantation, immune reconstitution, T-cell depleted graft, transplant outcomes

## Abstract

**Introduction:**

Immune reconstitution (IR) after allogeneic stem cell transplantation has been highlighted as pivotal in achieving favorable long-term outcomes by influencing the rates of infection, graft versus host disease (GvHD) and relapse. However, data on the impact of different lymphocyte subsets influencing outcomes is conflicting. Furthermore, the importance of immune reconstitution parameters in patients previously not experiencing major post-transplant complications is lacking.

**Methods:**

We evaluated the clinical impact of day 90 NK cell, CD4^+^ T-cell, CD8^+^ T-cell, B-cell, and NKT cell counts on transplant outcomes by performing a landmark analysis in event-free patients. Lymphocyte subset counts were obtained from 70 patients undergoing in vivo T-cell depleted allogeneic transplantation from 2018 to 2024. Patients eligible for the study experienced no acute GvHD, poor graft function, graft failure, or relapse in the first three months after transplantation-prior to obtaining IR data. We associated lymphocyte subset counts to overall survival (OS), non-relapse mortality (NRM), cumulative incidence of relapse (RI), and secondary graft failure/poor graft function.

**Results:**

High NK cell counts on day 90 (>178/μL) were associated with improved OS (P=0.039) and lower rates of NRM (1-year cumulative incidence of 5.7% versus 31.4%, HR 0.16, 95% CI 0.04-0.69, P=0.014). A protective effect on RI was not found. We found no patient, disease or transplant-related variables to be significantly associated with day 90 NK cell counts.

**Conclusion:**

The results suggest that high NK cell counts on day 90 after T-cell depleted allogeneic transplantation independently protect from NRM and improve OS in patients without prior major post-transplant complications.

## 1 Introduction

Allogeneic stem cell transplantation (allo-SCT) is a potentially curative treatment option for adult patients with high-risk hematological diseases (1). The success of allo-SCT is majorly influenced by the rates of opportunistic infections, disease relapse, and graft versus host disease (GvHD).

Immune reconstitution (IR), defined as a timely reconstitution of a donor-derived immune system, has been highlighted as pivotal in achieving favorable long-term outcomes by influencing the three most common complications mentioned above. Severe immune deficiency in the post-transplant period results in a prolonged susceptibility to infections (2, 3). Additionally, imbalances in IR can result in insufficient control of residual disease, leading to relapse, whereas impaired T-cell tolerance contributes to the development of GvHD (4).

The first studies evaluating the impact of immune reconstitution on transplant outcomes focused on the absolute lymphocyte counts (ALC) (5–7). Since then, the focus has been switched to lymphocyte subsets, with many studies correlating high CD4^+^ T-cell counts to improved outcomes, both in T-replete and T-deplete models (3, 8–14). However, other studies linked NK cell recovery to improved transplant outcomes, with variable time points at which NK cell counts proved to be beneficial (15–22). Finally, the importance of CD8^+^ T-cells was occasionally reported (23, 24). Recently, a series of studies done in pediatric and young adult patients defining CD4^+^ T-cell IR as two consecutive measurements of >50/µL in the first 100 days showed improved OS, and lower rates of NRM and relapse (25–30). Neither of the previously mentioned studies stated to exclude patients experiencing acute GvHD grade II-IV, poor graft function or secondary graft failure before or during the measurement of IR data, all of which most frequently occur in the first three months after transplantation (31, 32). These events predispose patients to worse outcomes and inherently affect immune reconstitution kinetics (8, 33). Acute GvHD decreases thymic output and thus recovery of CD4^+^ T-cells, while corticosteroids as first-line treatment further promote lymphopenia (12). Additionally, majority of these studies extrapolated IR cut-off values from the literature, even though these are still not standardized, resulting in a wide range of different IR definitions used.

Therefore, our goal was to investigate the impact of IR in patients not experiencing major events in the first three months after transplantation. We performed a landmark analysis in event-free patients defined as patients who did not experience acute GvHD grade II-IV, disease relapse, graft failure, or poor graft function in the first three months after transplantation. We aimed to test if CD4^+^ T-cells would emerge as predictors for favorable outcomes, as suggested by the most recent studies focused on the first 100 days post-transplantation, or if it perhaps would be the NK cells, since their protective effect was previously demonstrated in variable intervals, ranging from one to twelve months after transplantation. We investigated the impact of immune reconstitution of CD4^+^ T-cells (CD4 IR) and NK cells (NK IR) on OS, NRM and relapse rates in our cohort of event-free patients. We also evaluated these outcomes in the context of CD8^+^ T-cell (CD8 IR), B-cell (CD19 IR) and NKT-cell immune reconstitution (NKT IR).

## 2 Materials and methods

### 2.1 Study design and patients

We performed a retrospective study of patients who underwent their first allo-SCT at Clinic of Hematology, University Clinical Center of Serbia, between August 2018 and July 2024. There was no restriction on the indication for transplantation. Data was prospectively collected in the institutional database. As previously mentioned, for this study, we included event-free patients defined as patients who were alive three months after transplantation and had not previously experienced acute GvHD grade II-IV, relapse, primary or secondary graft failure, or poor graft function. We obtained IR data by measuring absolute counts of lymphocyte subpopulations on day 90 and divided patients into two groups based on the median value, with IR of different subpopulations defined as values above the median. We then performed a landmark analysis from day 90 after transplantation and correlated the two groups to different clinical outcomes. Institutional Review Board (IRB) approved this retrospective research protocol (IRB 307/6). All patients had previously given consent for the use of their medical records and blood analyses for research purposes. For this analysis the need for a specific informed consent was waived.

### 2.2 Procedures

All patients received conventional allo-SCT with peripheral blood as a stem cell source (PBSCT). Matched sibling donor (MSD) recipients received GvHD prophylaxis with cyclosporine A (3mg/kg from day -1) and methotrexate (15mg/m^2^ on day 1, and 10mg/m^2^ on day 3 and day 6). Matched unrelated donor (MUD) and mismatched unrelated donor (MMUD) recipients received the same prophylaxis with an additional dose of methotrexate (10mg/m^2^) on day 11. MSD, MUD and MMUD patients received *in vivo* T-cell depletion (TCD) with anti-thymocyte globulin (Grafalon^®^) 10mg/kg from day -3 until day -1 for MSD and 20mg/kg from day -3 until day -1 for MUD and MMUD. Patients undergoing haploidentical transplantation received GvHD prophylaxis with cyclosporine A (3mg/kg from day -1) and mycophenolate mofetil (15mg/kg three times daily from day 5 until day 28) and *in vivo* TCD with posttransplant cyclophosphamide (50mg/kg/day on d+3 and d+4). CMV prophylaxis with letermovir in CMV seropositive recipients was started in November 2019. All patients received chemotherapy-based conditioning regimens and were treated in high-efficiency, positive-pressure isolation rooms. Patients received gut decontamination with fluoroquinolones and infection prophylaxis against HSV, VZV, PCP and fungi according to local protocols.

### 2.3 Flow cytometry and lymphocyte subset enumeration

Lymphocyte subsets were measured by flow cytometry on day 90 after transplantation on EDTA-treated whole blood. An aliquot of 100 - 150 µl of the whole peripheral blood was incubated with a combination of seven monoclonal antibodies at saturated concentrations: fluorescein isothiocyanate-conjugated anti-CD3 (Clone SK7; Becton Dickinson), phycoerythrin-conjugated anti-CD16 (Clone B73.1; Becton Dickinson), phycoerythrin-conjugated anti-CD56 (Clone NCAM16.2; Becton Dickinson), peridinin- chlorophyll protein-conjugated anti-CD4 (Clone SK3; Becton Dickinson), allophycocyanin-conjugated anti-CD19 (Clone SJ25C1; Becton Dickinson), phycoerythrin-Cyanin7-conjugated anti-CD8 (Clone SFCI21Thy2D3; Beckman Coulter), and V500-conjugated anti-CD45 (Clone HI30; Becton Dickinson). After incubation, erythrocyte lysis was performed by commercial lysis reagent according to protocol (FACS Lysing Solution; Becton Dickinson). Analysis was performed on BD FACSCanto II (4-2-2) (Becton Dickinson, USA) using BD FACSDiva Software ver 8.0.1/ver 9.0.1 (Becton Dickinson, USA). The flow cytometer was aligned daily with a standard fluorospheres set (BD FACSDiva CS&T IVD Beads; Becton Dickinson, USA) and BD CS&T Software ver 3.0 (Becton Dickinson, USA). Analysis of lymphocyte subsets, based on immunophenotype characteristics, included T helper cells (CD3^+^CD4^+^), cytotoxic T cells (CD3^+^CD8^+^), B-cells (CD19^+^), NK-cells (CD3^-^CD16^+^CD56^+^), and NKT-cells (CD3^+^CD16^+^CD56^+^) (34). The absolute numbers of lymphocyte subsets were calculated by using double-platform (34). External quality control of the applied assay was performed periodically by using BD Multi-Check CD4 Low Control (Becton Dickinson, USA).

### 2.4 Outcomes

Landmark survival analyses at three months were performed to assess the association between immune reconstitution factors and clinical outcomes. The main outcomes of interest were overall survival (OS) and the incidence of NRM in relation to achieving CD4, NK, CD8, CD19 and NKT IR. Secondary outcomes of interest were event-free survival (EFS), cumulative incidence of relapse (RI), acute GvHD grade II-IV, and secondary graft failure/poor graft function (sGF/PGF).

OS was defined as time from transplantation until death or last follow-up. NRM was defined as death in the absence of relapse. For patients with AML, ALL, and MDS, relapse was defined as the presence of more than 5% blast cells in the bone marrow. In patients with Hodgkin’s lymphoma, relapse was defined by the appearance of new lesions or increase by ≥50% of previously involved sites from nadir, based on imaging studies. Patients with aplastic anemia were excluded from the relapse analysis. EFS was defined as survival from transplantation until disease relapse, occurrence of acute GvHD grade II-IV, extensive chronic GvHD, sGF/PGF, death or last follow-up, whichever occurred first. sGF/PGF was defined as time from transplantation until secondary graft failure or poor graft function. Graft failure and poor graft function were defined according to harmonized definitions (35). Acute GvHD (grade II–IV) was classified according to the Glucksberg criteria (36). Chronic GvHD (extensive *vs* no or limited) was classified according to the Shulman criteria (37) because the retrospective nature of the study precluded accurate severity scoring per consensus criteria in all patients. All surviving patients were censored at the date of last contact.

### 2.5 Statistical analysis

The results were analyzed according to the information available as of December 1^st^ 2024. The impact of lymphocyte reconstitution on outcomes was analyzed in univariate manner by categorizing subset counts as dichotomous variables according to the median value and calculating probabilities of OS and EFS using Kaplan–Meier model. We evaluated the probabilities of NRM, relapse, acute GvHD grade II-IV and sGF/PGF using cumulative incidence models. Estimated survival and cumulative incidence rates were compared by log-rank and Gray’s test, respectively. For NRM, relapse was a competing event; for relapse, NRM was a competing event; for acute GvHD, death or relapse were competing events, for sGF/PGF, death not caused by sGF/PGF was a competing event. Additionally, a univariable Cox proportional hazard model (PH) was performed on each lymphocyte subpopulation, with IR data assessed as a categorical variable, along with other patient, disease and transplant-related variables. If a significant association was observed between the IR of a specific lymphocyte subpopulation and a transplant outcome (*P*<0.05), a multivariate (MV) Cox PH model was subsequently constructed using forward stepwise selection. In addition to IR data, other variables considered for MV analysis, based on clinical relevance, were: age at transplant, early CMV reactivation, EBMT risk score and conditioning regimen intensity.

We then evaluated the impact of different patient, disease and transplant-related variables on achieving CD4 IR, NK IR, CD8 IR, CD19 IR and NKT IR. Variables considered for the analysis were: patient age at transplant (≤/>median age of 39), diagnosis (AML vs ALL vs MDS vs other; myeloid vs lymphoid), disease status at transplant (CR1, CR2 or active disease), ECOG PS score (≤/>1), HCT-CI score (≤/>1), donor-patient sex match, donor-patient CMV status match, minimal residual disease (MRD) status at transplant (positive vs negative), EBMT risk score (≤/>2), type of transplant (MSD vs MUD vs MMUD vs haploidentical), conditioning regimen (myeloablative or reduced intensity), number of infused CD34^+^ cells (≤/> median), use of ATG or post-transplant cyclophosphamide for *in vivo* TCD, MRD status at day 30 after transplantation (positive vs negative), CMV prophylaxis with letermovir (only in CMV seropositive recipients), occurrence of severe bacterial infections (defined as fever with positive blood cultures in the first three months after transplantation), and early CMV reactivation (defined as positive quantitative CMV PCR in two consecutive blood samples with more than 100 copies/mL, in the first three months after transplantation). Univariate associations were assessed using the Chi-square test, while multivariate analysis was performed using logistic regression. Finally, the following variables were analyzed in the multivariate model: age at transplant, type of transplant, donor-patient sex match, donor-patient CMV match, early CMV reactivation and EBMT risk score. Statistical analyses were done using IBM SPSS version 21.0.0., with a *P* value of <0.05 considered statistically significant.

## 3 Results

### 3.1 Patient characteristics

Among 150 patients that were transplanted in our Clinic in the period between August 2018 and July 2024, we identified 76 event-free patients who were eligible for the study. Six patients did not have lymphocyte subpopulation counts at day 90 and were therefore excluded from the analysis, with a dropout rate of 8.5%. Patient and transplantation characteristics are shown in **Table 1**. Median age at transplant was 39 years (range 18-61), with equal distribution of male and female patients (N=35). The most frequent indication for transplantation was AML (N= 32, 45.7%), followed by ALL (N=21, 30%). Most of the patients received myeloablative conditioning (N=49, 70%). MUD was the most common type of donor (N=30, 42.9%), followed by MSD (N=27, 38.6%), MMUD (N=9, 12.8%) and haploidentical (N=4, 5.7%). The median day 90 absolute count of CD4^+^ T-cells, NK cells, CD8^+^ T-cells, B-cells, and NKT cells was 56/µL, 178/µL, 160/µL, 9/µL and 40/µL, respectively (**Table 1**).

**Table 1:** Patient, disease, and transplant characteristics and absolute lymphocyte subpopulation counts.

Patient characteristics	N(%)
Number of patients	70
Male sex	35 (50)
Median age at transplant (range)	39 (18-61)
Diagnosis	
AML	32 (45.7)
ALL	21 (30)
Myelodysplastic syndrome	8 (11.5)
Hodgkin’s lymphoma	5 (7.1)
Severe aplastic anemia	3 (4.3)
Primary myelofibrosis	1 (1.4)
Disease status (AML and ALL only)	
CR1	40 (75.5)
CR2	8 (15)
Active disease	5 (9.5)
HCT CI	
0-1	66 (94.2)
>1	4 (5.8)
EBMT risk score	
0-2	29 (42.65)
>2	39 (57.35)
Recipient/Donor CMV serostatus	
-/-	5 (7.1)
-/+	8 (11.4)
+/-	24 (34.3)
+/+	33 (47.2)
Prophylaxis with letermovir* (yes)	41 (72)
Donor type	
MSD	27 (38.6)
MUD	30 (42.9)
MMUD	9 (12.8)
Haploidentical	4 (5.7)
Recipient/Donor sex	
Male/Male	23 (32.9)
Male/Female	12 (17.1)
Female/Male	24 (34.3)
Female/Female	11 (15.7)
Conditioning intensity	
MAC	49 (70)
RIC	21 (30)
*In vivo TCD*	
ATG	66 (94)
Post-Cy	4 (6)
Infused CD34^+^ cells (median, IQR)**	7.08 (5.03-8.94)
Severe bacterial infections (no)	16 (22.8)
Early CMV reactivation (yes)	27 (38.6)
Positive MRD pretransplant (yes)	32 (72.7)
Positive MRD on day 30	22 (42.3)
Day 90 CD4^+^ T-cell count/µL (median, range)	56 (2-315)
Day 90 NK cell count/µL (median, range)	178 (8-823)
Day 90 CD8^+^ T-cell count/µL (median, range)	160 (0.5-2323)
Day 90 B-cell count/µL (median, range)	9 (0-380)
Day 90 NKT cell count/µL (median, range)	40 (1-555)

Values are n (%) of patients unless indicated otherwise.

*only in seropositive patients.

**expressed as n x10^6^/kg.

AML, acute myeloid leukemia; ALL, acute lymphoblastic leukemia; CR1, first clinical remission; CR2, second clinical remission; MAC, myeloablative conditioning; MMUD, mismatched unrelated donor; MRD, minimal residual disease; MSD, matched sibling donor; MUD, matched unrelated donor; RIC, reduced intensity conditioning.

### 3.2 Transplant outcomes

After a median follow-up of 18.5 months, 50 out of 70 patients are alive (71,4%). Seven patients (10%) died due to relapse and thirteen (18.6%) due to NRM. Causes of NRM were acute GvHD in four cases, infections in seven cases, and poor graft function or secondary graft failure with one case each. Deaths due to infections were caused by viral pathogens, with three cases caused by SARS-CoV-2 and CMV respectively. Six patients experienced acute GvHD grade II-IV during the tapering of immunosuppression, with two additional cases occurring after the administration of DLI while treating relapse. It is worth mentioning that therapeutic DLI was administered after day 90, therefore, it did not impact IR dynamics. Additionally, patients experiencing GvHD after receiving DLI were excluded from the analysis of acute GvHD as an endpoint, since they had already relapsed, which was a competing event by study design. Furthermore, no prophylactic or preemptive DLI was administered before day 90 in our study cohort. Only one patient from the entire cohort previously having acute GvHD developed extensive chronic GvHD (and interestingly experienced relapse after this.

**Figure 1:**
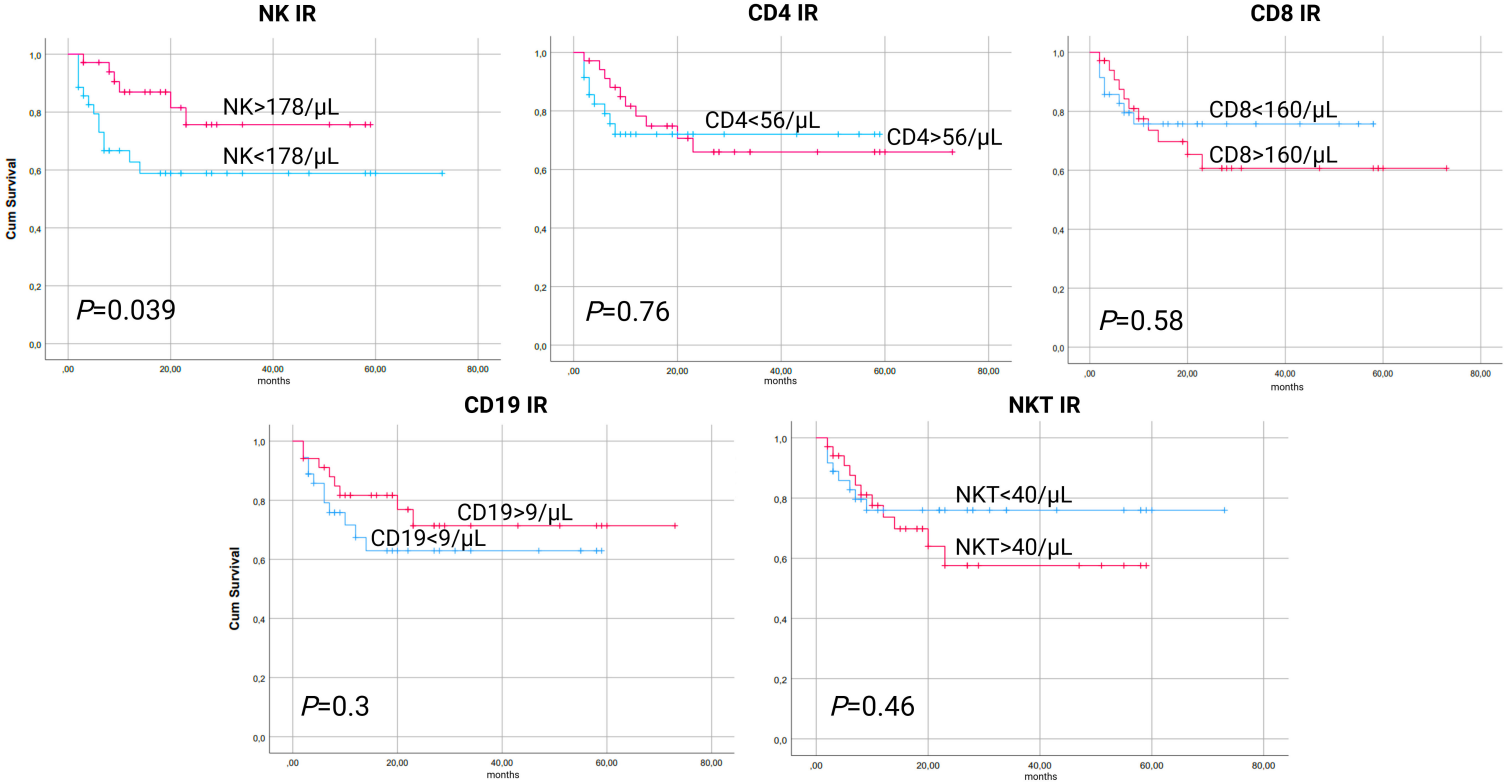
Kaplan-Meier curves for overall survival (OS) according to lymphocyte subpopulation counts on day 90 after transplantation. CD4-CD4^+^ T-cell, CD8-CD8^+^ T-cell, CD19-B-cell, IR-immune reconstitution, NK-NK cell, NKT-NKT cell.

NK IR, defined as NK cell count above the median of 178/µL, was associated with improved OS (*P*=0.039, **Figure 1**). CD4 IR, CD8 IR, CD19 IR and NKT IR did not show any statistically significant associations with OS (**Figure 1**).

**Figure 2:**
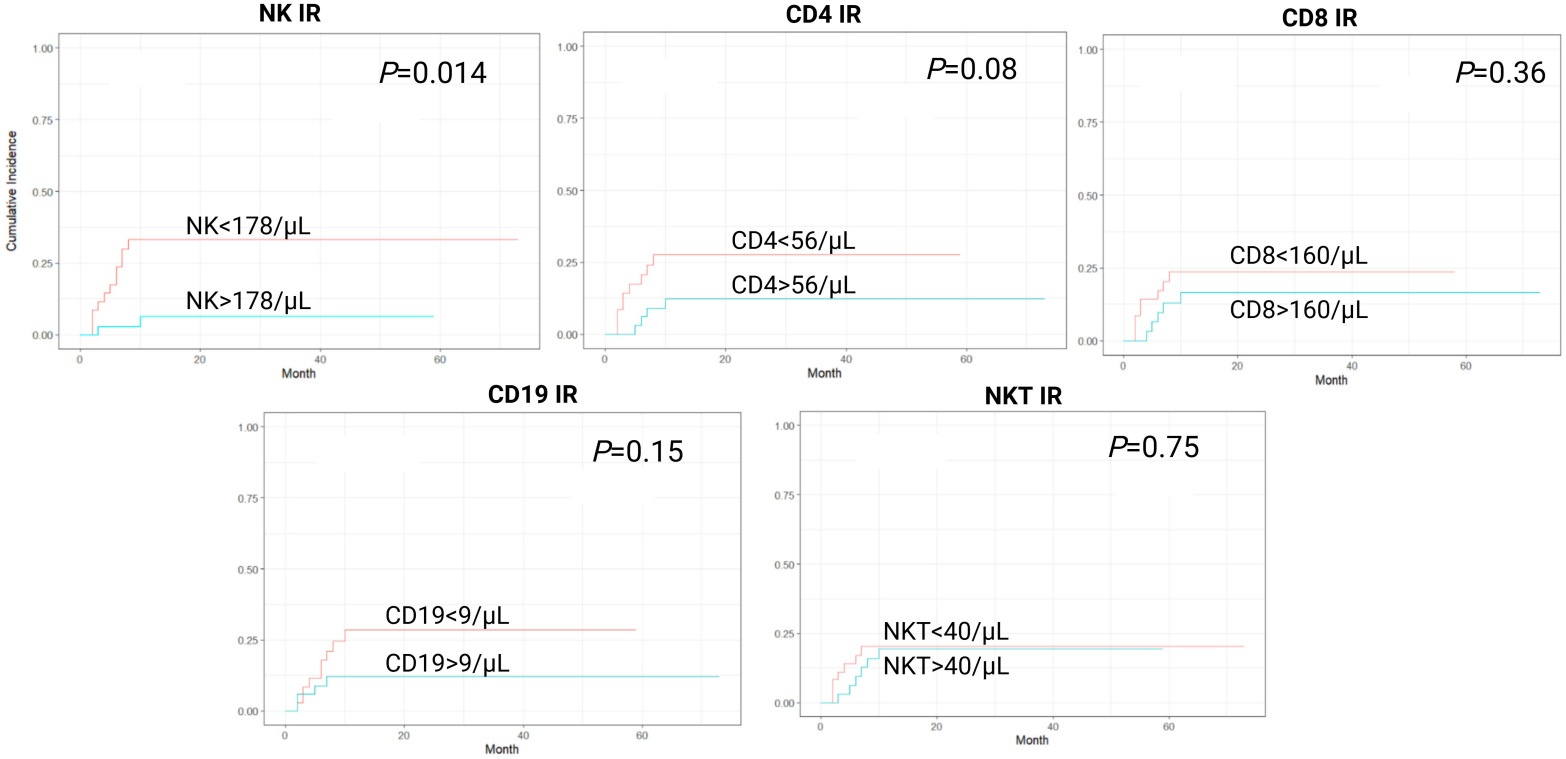
Cumulative incidence curves for non-relapse mortality (NRM) according to lymphocyte subpopulation counts on day 90 after transplantation. CD4-CD4^+^ T-cell, CD8-CD8^+^ T-cell, CD19-B-cell, IR-immune reconstitution, NK-NK cell, NKT-NKT cell.

**Figure 3:**
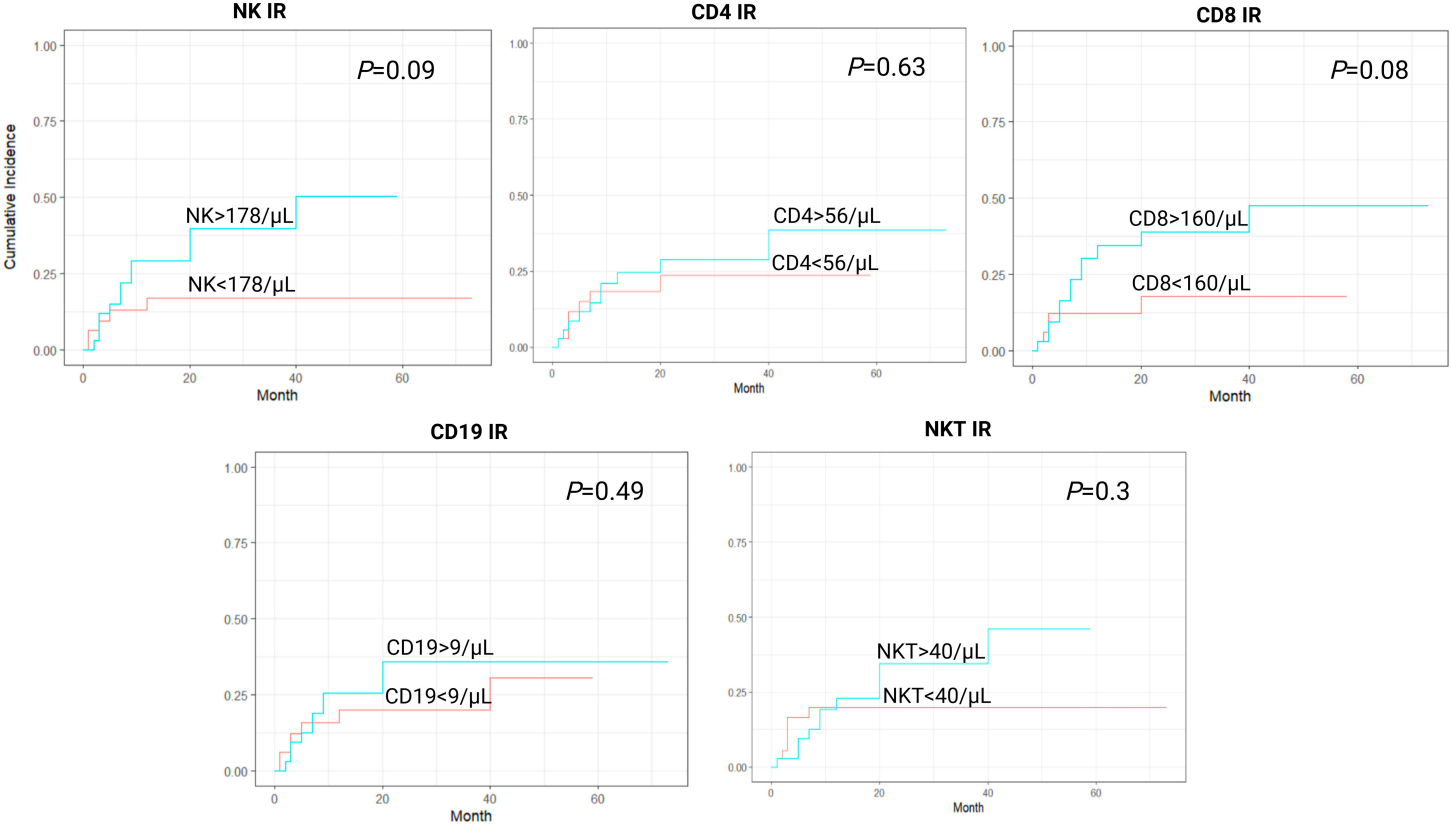
Cumulative incidence curves for relapse incidence (RI) according to lymphocyte subpopulation counts on day 90 after transplantation. CD4-CD4^+^ T-cell, CD8-CD8^+^ T-cell, CD19-B-cell, IR-immune reconstitution, NK-NK cell, NKT-NKT cell.

**Figure 4:**
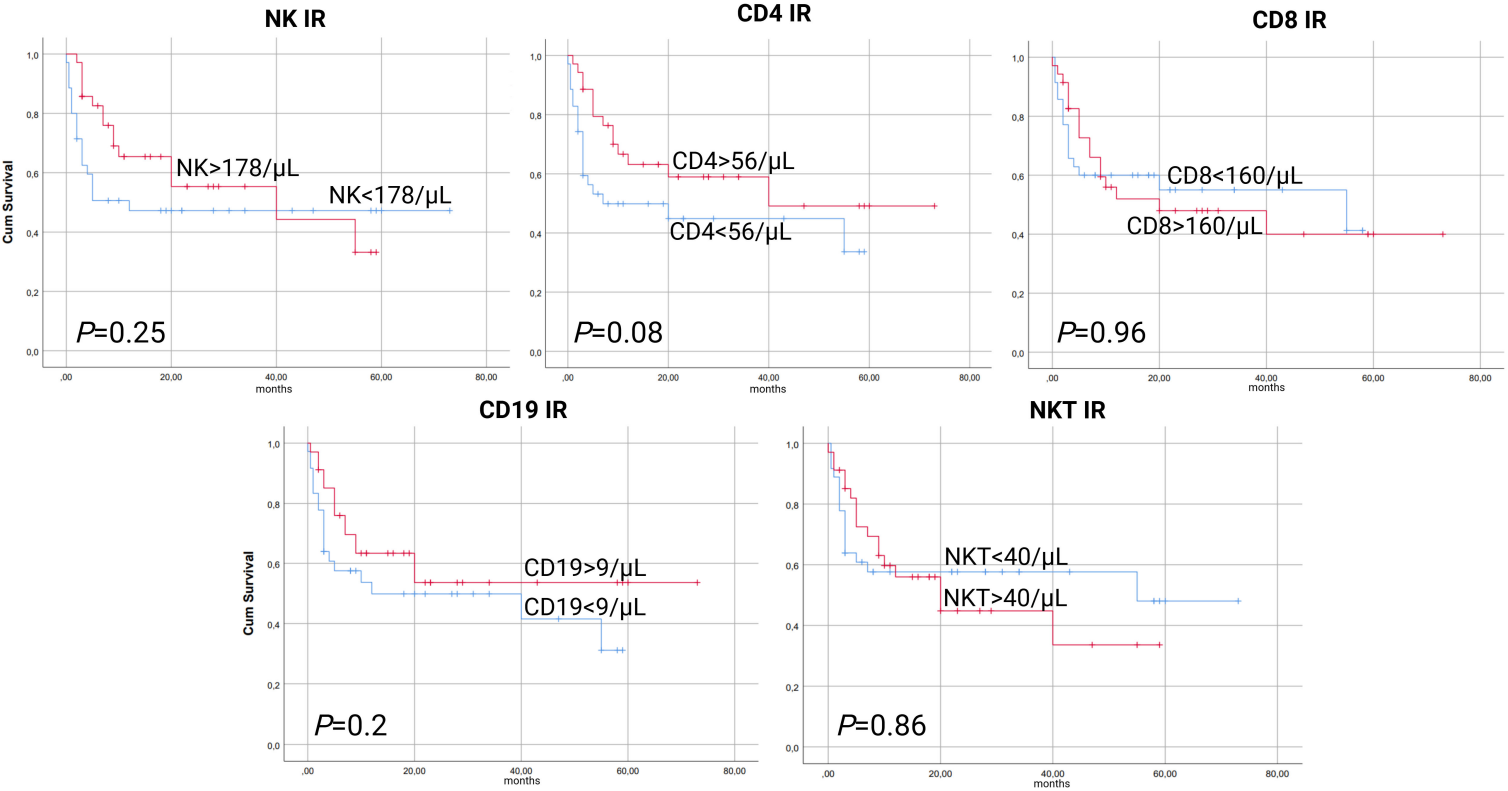
Kaplan-Meier curves for event-free survival (EFS) according to lymphocyte subpopulation counts on day 90 after transplantation. CD4-CD4^+^ T-cell, CD8-CD8^+^ T-cell, CD19-B-cell, IR-immune reconstitution, NK-NK cell, NKT-NKT cell.

**Figure 5:**
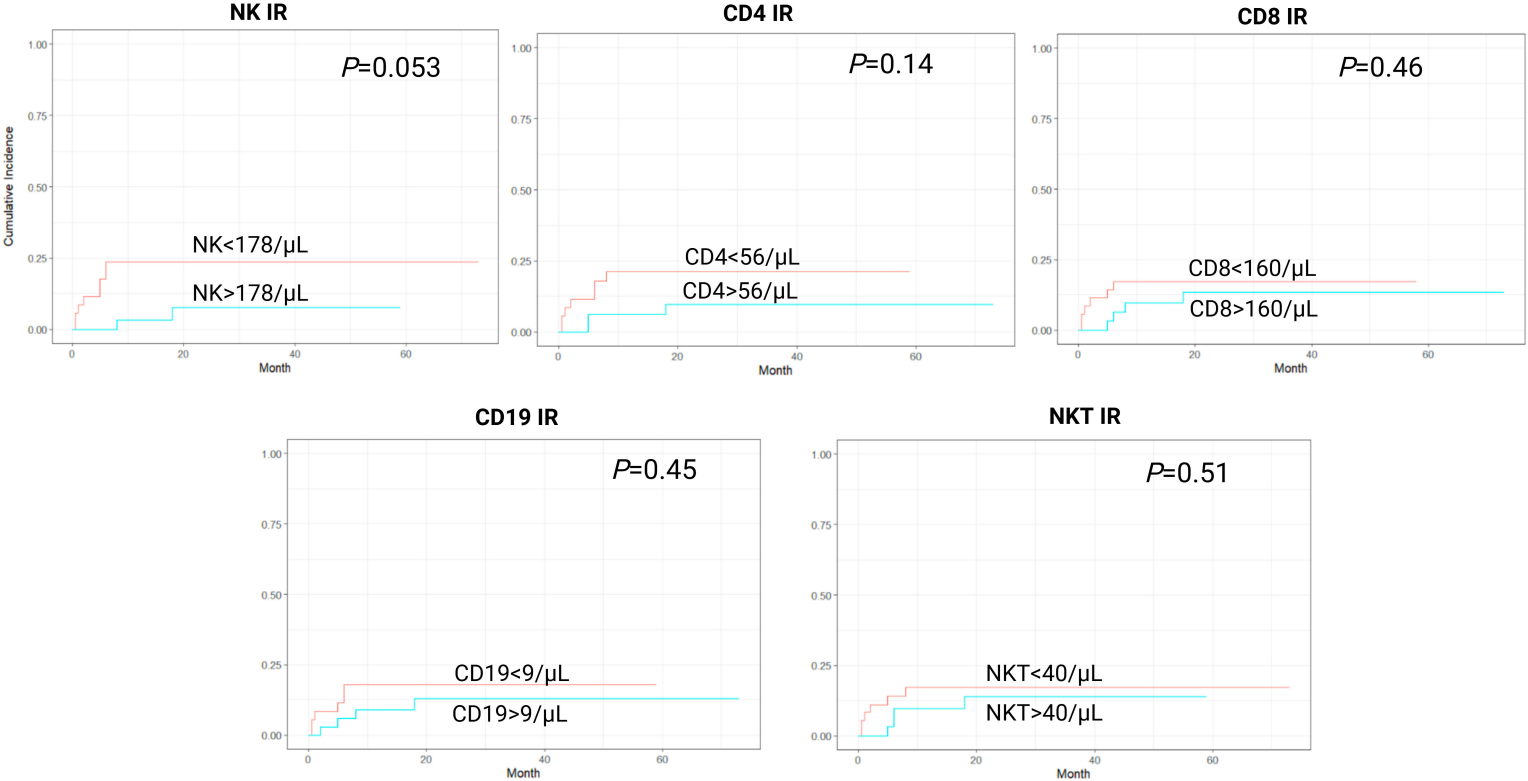
Cumulative incidence curves for secondary graft failure/poor graft function (sGF/PGF) according to lymphocyte subpopulation counts on day 90 after transplantation. CD4-CD4^+^ T-cell, CD8-CD8^+^ T-cell, CD19-B-cell, IR-immune reconstitution, NK-NK cell, NKT-NKT cell.

**Figure 6:**
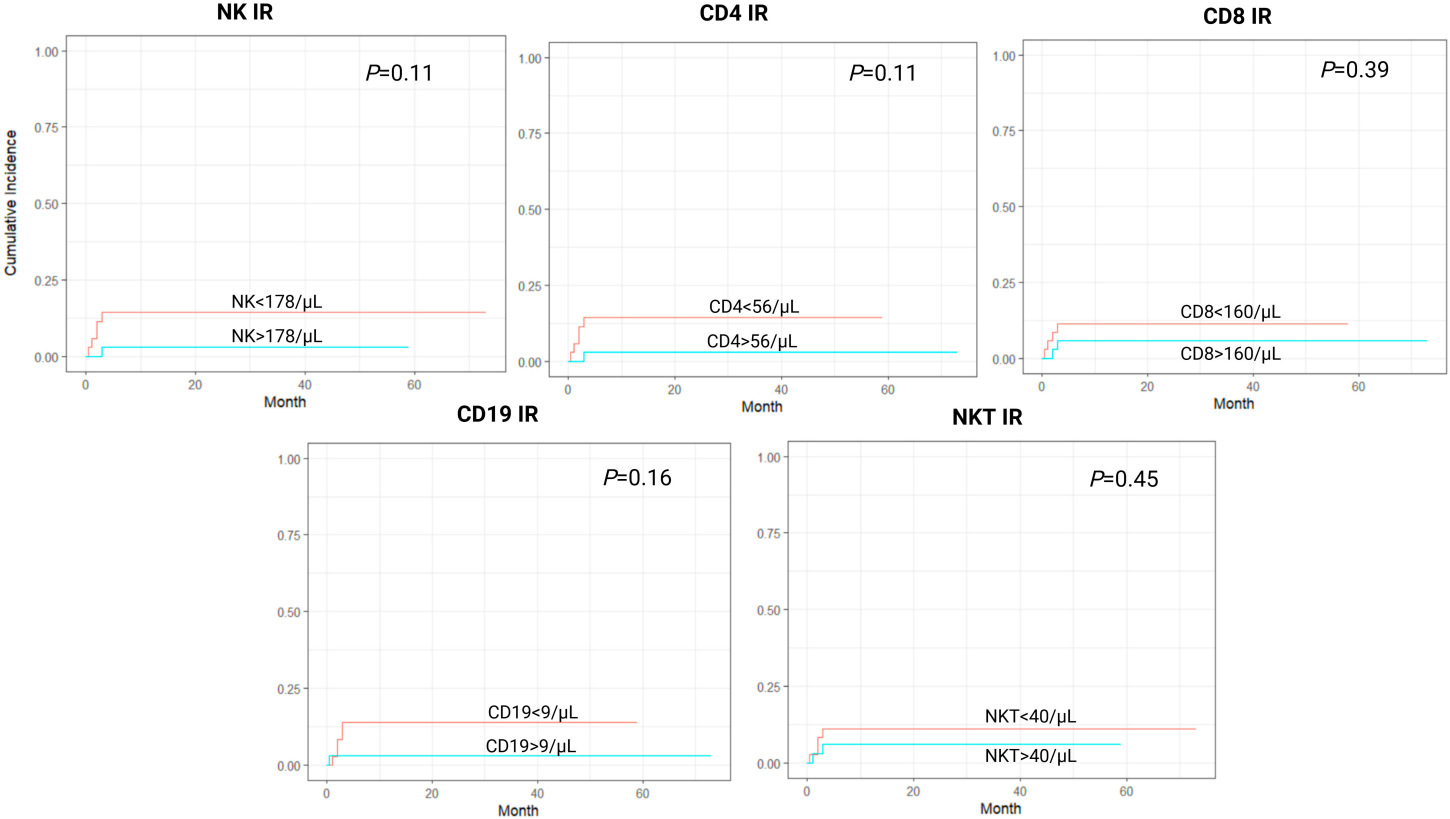
Cumulative incidence curves for acute graft-versus-host disease grade II-IV (aGvHD gr II-IV) according to lymphocyte subpopulation counts on day 90 after transplantation. CD4-CD4^+^ T-cell, CD8-CD8^+^ T-cell, CD19-B-cell, IR-immune reconstitution, NK-NK cell, NKT-NKT cell.

In a cumulative incidence model, NK IR was also associated with significantly lower rates of NRM, with 1-year NRM rate of 5.7% versus 31.4% in patients with and without NK IR, respectively (HR 0.16, 95% CI 0.04-0.69, *P*=0.014). Additionally, patients with CD4 values above the median of 56/µL had lower rates of NRM, although this was not proven to be statistically significant (HR 0.37, 95% CI 0.12-1.15, *P*=0.08). No association between CD8, CD19 or NKT IR and NRM was found (**Figure 2**). Cumulative incidence of relapse analysis did not show any statistically significant association between IR and relapse rates (**Figure 3**). However, patients achieving NK and CD8 IR showed a trend toward higher relapse rates (35% vs 16%, *P*=0.09 and 36% vs 15%, *P=0.08*, respectively). We further analyzed relapse rates in a subgroup of patients with myeloid malignancies, but did not find any statistically significant associations (data not shown). Thirty-three patients (47%) experienced an event (relapse, death, graft failure, acute GvHD grade II-IV or extensive chronic GvHD). No associations between NK IR, CD4 IR, CD8 IR, CD 19 IR, and NKT IR with EFS were found, although patients with CD4 IR showed trends toward higher EFS (*P*=0.084, **Figure 4**). Ten patients (14.3%) experienced secondary graft failure or poor graft function. NK IR was associated with lower rates of sGF/PGF (22% vs 5.7%) with a borderline statistical significance (HR 0.23, 95% CI 0.05-1.02, *P*=0.053). Other lymphocyte subpopulations were not associated with the occurrence of sGF/PGF (**Figure 5**). As previously mentioned, six patients (8.6%) experienced acute GvHD grade II-IV, when excluding two cases where it resulted as a complication of DLI administration. There was a trend toward higher rates of acute GvHD in patients without NK or CD4 IR (14% vs 3%, *P*=0.11), however, the limited number of events precluded definite conclusions (**Figure 6**).

Univariate Cox PH model evaluating the impact of each lymphocyte subpopulation and other variables on transplant outcomes is shown in **Tables 2**–**4**. In univariate analysis, NK IR was significantly associated with improved OS, reduced rates of NRM, and reduced rates of sGF/PGF. This association was preserved in multivariate analysis, as NK IR was independently associated with improved overall survival (HR 0.38, 95% CI 0.14-0.99, *P*=0.049), decreased incidence of NRM (HR 0.16, 95% CI 0.04-0.73, *P=*0.018) and decreased incidence of sGF/PGF (HR 0.2, 95% CI 0.04-0.96, *P*=0.04). Other covariates included in the analysis (age at transplant, early CMV reactivation, conditioning regimen intensity, EBMT risk score) did not show significant associations with these outcomes (**Table 5**). The association between CD4 IR, CD8 IR, CD19 IR and NKT IR with OS, NRM and sGF/PGF was not demonstrated, and neither lymphocyte subpopulation was significantly associated with RI, acute GvHD grade II-IV, and EFS.

**Table 2:** Univariable Cox proportional hazard analysis for overall survival (OS) and non-relapse mortality (NRM).

	OS			NRM		
	HR	95% CI	*P* value	HR	95% CI	*P* value
Gender (female)	1.05	0.42-2.58	0.92	0.81	0.27-2.41	0.7
Age (≥median)	0.76	0.3-1.92	0.562	1.4	0.47-4.17	0.54
Diagnosis (myeloid)	1.42	0.54-3.76	0.47	3	0.67-13.68	0.15
Disease status (CR)	0.46	0.1-2.1	0.31	0.49	0.06-3.93	0.5
ECOG PS (>1)	1.3	0.17-9.8	0.8	1.26	0.2-5.1	0.64
HCT CI (>1)	1.79	0.41-7.78	0.43	1.44	0.18-11.1	0.72
EBMT risk score (>2)	0.72	0.3-1.76	0.46	1.4	0.46-4.3	0.55
Sex match (Female donor-male recipient)	2.18	0.7-6.77	0.17	2.8	0.85-9.2	0.09
CMV serostatus (IgG^+^ donor/IgG^-^ recipient)	0.87	0.33-2.3	0.78	0.83	0.25-2.69	0.75
Donor type (MSD)	0.85	0.33-2.17	0.73	0.4	0.11-.1.46	0.17
Conditioning regimen (RIC)	0.38	0.1-1.3	0.12	0.89	0.27-2.89	0.84
Infused CD34^+^ (≥median)	0.55	0.21-1.44	0.23	0.39	0.12-1.27	0.12
ATG vs PTCy (ATG)	0.43	0.1-1.88	0.26	0.56	0.07-4.29	0.57
Severe bacterial infections (yes)	2.14	0.8-5.6	0.13	1.9	0.6-6.42	0.26
Early CMV reactivation (yes)	1.55	0.63-3.81	0.34	1.37	0.46-4.08	0.57
NK IR (>178/µL)	**0.37**	**0.14-0.98**	**0.048**	**0.16**	**0.0.4-0.73**	**0.017**
CD4 IR (>56/µL)	0.75	0.35-2.14	0.87	0.35	0.11-1.16	0.08
CD8 IR (>160/µL)	0.59	0.52-3.2	1.28	0.62	0.2-1.89	0.4
CD19 IR (>9/µL)	0.32	0.25-1.56	0.63	0.41	0.13-1.33	0.14
NKT IR (>40/µL)	0.46	0.56-3.5	1.4	0.77	0.26-2.3	0.64

*P* values less than 0.05 are bolded.

ATG, anti-thymocyte globulin; CI, confidence interval; CMV, cytomegalovirus: CR, complete remission; EBMT risk score, European Society for Blood and Marrow Transplantation risk score; ECOG PS, Eastern Cooperative Oncology Group Performance status; HCT-CI, Hematopoietic cell transplantation-specific comorbidity index; HR, hazard ratio; MSD, matched sibling donor: PTCy, post-transplant cyclophosphamide; RIC, reduced intensity conditioning.

**Table 3:** Univariable Cox proportional hazard analysis for secondary graft failure/poor graft function (sGF/PGF) and acute graft-versus-host disease (GvHD) grade II-IV.

	*sGF/PGF*			Acute GvHD grade II-IV		
	HR	95% CI	*P* value	HR	95% CI	*P* value
Gender (female)	1.46	0.41-5.18	0.56	1.03	0.2-5.1	0.97
Age (≥median)	1.96	0.55-6.96	0.29	1.18	0.24-5.86	0.84
Diagnosis (myeloid)	0.77	0.2-2.88	0.7	0.58	0.12-2.87	0.5
Disease status (CR)	0.41	0.05-3.48	0.41	0.32	0.04-2.91	0.31
ECOG PS (>1)	1.42	0.4-5.5	0.68	1.3	0.33-4.8	0.77
HCT CI (>1)	2.05	0.26-16.2	0.5	3.37	0.39-28.89	0.27
EBMT risk score (>2)	1.16	0.33-4.12	0.82	1.55	0.28-8.44	0.61
Sex match (Female donor-male recipient)	2.92	0.74-11.5	0.13	0.95	0.11-8.12	0.96
CMV serostatus (IgG^+^ donor/IgG^-^ recipient)	1.26	0.36-4.48	0.72	1.02	0.19-5.55	0.99
Donor type (MSD)	0.95	0.27-3.37	0.93	0.45	0.11-1.9	0.26
Conditioning regimen (RIC)	0.5	0.1-2.39	0.39	0.42	0.05-3.58	0.43
Infused CD34^+^ (≥median)	0.63	0.17-2.23	0.47	1	0.2-4.94	1
ATG vs PTCy (ATG)	0.7	0.09-5.1	0.64	0.27	0.03-2.3	0.23
Severe bacterial infections (yes)	1.84	0.47-7.16	0.38	1.86	0.34-10.18	0.47
Early CMV reactivation (yes)	2.64	0.75-9.37	0.13	3.55	0.65-19.4	0.14
NK IR (>178/µL)	**0.2**	**0.04-0.95**	**0.044**	0.17	0.02-1.49	0.11
CD4 IR (>56/µL)	0.35	0.09-1.37	0.13	0.18	0.02-1.56	0.12
CD8 IR (>160/µL)	0.63	0.18-2.23	0.47	0.47	0.08-2.55	0.38
CD19 IR (>9/µL)	0.58	0.16-2.05	0.4	0.2	0.02-1.75	0.15
NKT IR (>40/µL)	0.66	0.19-2.36	0.53	0.5	0.09-2.76	0.43

*P* values less than 0.05 are bolded.

ATG, anti-thymocyte globulin; CI, confidence interval; CMV, cytomegalovirus; CR, complete remission; EBMT risk score, European Society for Blood and Marrow Transplantation risk score; ECOG PS, Eastern Cooperative Oncology Group Performance status; HCT-CI, Hematopoietic cell transplantation-specific comorbidity index; HR, hazard ratio; MSD, matched sibling donor; PTCy, post-transplant cyclophosphamide; RIC, reduced intensity conditioning.

**Table 4:** Univariable Cox proportional hazard analysis for relapse incidence (RI) and event-free survival (EFS).

	RI			EFS		
	HR	95% CI	*P* value	HR	95% CI	*P* value
Gender (female)	0.77	0.3-2	0.6	0.86	0.42-1.72	0.66
Age (≥median)	0.61	0.28-1.67	0.34	0.8	0.39-1.62	0.54
Diagnosis (myeloid)	0.4	0.15-1.2	0.07	0.8	0.39-1.63	0.54
Disease status (CR)	0.46	0.1-2.1	0.32	0.57	0.17-1.92	0.37
ECOG PS (>1)	3.45	0.78-15.2	0.11	1.83	0.43-7.67	0.41
HCT CI (>1)	1.8	0.43-8.24	0.4	1.62	0.49-5.33	0.42
EBMT risk score (>2)	1.76	0.62-5	0.29	1.3	0.64-2.68	0.46
Sex match (Female donor-male recipient)	0.4	0.05-3.1	0.39	1.22	0.47-3.21	0.68
CMV serostatus (IgG^+^ donor/IgG^-^ recipient)	1.34	0.5-3.52	0.55	1.19	0.58-2.44	0.63
Donor type (MSD)	1.45	0.55-3.82	0.45	0.73	0.35-1.52	0.4
Conditioning regimen (RIC)	1.3	0.45-3.4	0.68	0.87	0.41-1.86	0.73
Infused CD34^+^ (≥median)	1.05	0.38-2.93	0.92	0.66	0.32-1.36	0.26
ATG vs PTCy (ATG)	1	0.13-7.61	1	0.87	0.2-3.64	0.85
Severe bacterial infections (yes)	0.97	0.28-3.38	0.96	1.2	0.52-2.79	0.67
Early CMV reactivation (yes)	0.89	0.33-2.4	0.81	1.37	0.68-2.76	0.38
NK IR (>178/µL)	2	0.7-5.72	0.2	0.63	0.31-1.26	0.19
CD4 IR (>56/µL)	1.05	0.4-2.79	0.91	0.58	0.29-1.17	0.13
CD8 IR (>160/µL)	2.4	0.8-6.8	0.11	1.05	0.52-2.1	0.89
CD19 IR (>9/µL)	1.27	0.48-3.34	0.63	0.68	0.34-1.37	0.28
NKT IR (>40/µL)	1.6	0.6-4.22	0.35	1.1	0.55-2.2	0.79

*P* values less than 0.05 are bolded.

ATG, anti-thymocyte globulin; CI, confidence interval; CMV, cytomegalovirus; CR, complete remission; EBMT risk score, European Society for Blood and Marrow Transplantation risk score; ECOG PS, Eastern Cooperative Oncology Group Performance status; HCT-CI, Hematopoietic cell transplantation-specific comorbidity index; HR, hazard ratio; MSD, matched sibling donor: PTCy, post-transplant cyclophosphamide; RIC, reduced intensity conditioning.

**Table 5:** Multivariable Cox proportional hazard analysis for overall survival (OS), non-relapse mortality (NRM) and secondary graft failure/poor graft function (sGF/PGF).

	OS	NRM	sGF/PGF
	*P* value	*P* value	*P* value
NK IR (>178/*µL)*	**0.049**	**0.018**	**0.04**
Age (>median)	0.63	0.43	0.27
Early CMV reactivation (yes)	0.44	0.8	0.18
Conditioning regimen (RIC)	0.14	0.97	0.47
EBMT risk score (>2)	0.42	0.62	0.91

CMV,cytomegalovirus; EBMT risk score, European Society for Blood and Marrow Transplantation risk score; RIC, reduced intensity conditioning.

### 3.3 Lymphocyte subset reconstitution predictors

The results of univariate analysis are shown in **Table 6**. Recipient CMV seropositivity was associated with both CD4^+^ and CD8^+^ T-cell count in univariate analysis. When compared to the other three combinations, CMV seropositive patients having seronegative donors had lower CD4^+^ T-cell counts, while seropositive patients having seropositive donors had higher CD8^+^ T-cell counts. Patients transplanted from MSD had higher CD8^+^ T-cell and NKT cell counts. Patients with lower EBMT risk score also had higher NKT cell counts.

**Table 6:** Univariate regression analysis on immune reconstitution.

	NK IR	CD4 IR	CD8 IR	CD19 IR	NKT IR
Gender (male)	NS	NS	NS	NS	NS
Age (>median)	NS	NS	NS	NS	NS
Diagnosis	NS	NS	NS	NS	NS
Disease status	NS	NS	NS	NS	NS
ECOG PS (>1)	NS	NS	NS	NS	NS
HCT-CI (>1)	NS	NS	NS	NS	NS
EBMT score (>2)	NS	NS	NS	NS	*P*=0.017^a^
Donor-patient sex match	NS	NS	NS	NS	NS
CMV serostatus	NS	*P*=0.004^b^	*P*=0.033^c^	NS	NS
MRD positivity at transplant	NS	NS	NS	NS	NS
Donor type	NS	NS	*P*=0.008^d^	NS	*P*=0.000^d^
Conditioning (MAC)	NS	NS	NS	NS	NS
Infused CD34^+^ cells (>median)	NS	NS	NS	NS	NS
ATG vs PTCy	NS	NS	NS	NS	NS
CMV prophylaxis	NS	NS	NS	NS	NS
Severe bacterial infections	NS	NS	NS	NS	NS
Early CMV reactivation	NS	NS	NS	NS	NS
MRD positivity at day 30	NS	NS	NS	NS	NS

a Higher counts in patients with a lower EBMT score.

b Lower counts in CMV seropositive patients with a seronegative donor.

c Higher counts in CMV seropositive patients with a seropositive donor.

d Higher counts in patients with a matched related donor.

ATG, anti-thymocyte globulin; EBMT score, European Society for Blood and Marrow Transplantation risk score; ECOG PS, Eastern Cooperative Oncology Group Performance status; HCT CI, Hematopoietic Cell Transplantation-specific Comorbidity Index; MAC, myeloablative conditioning; MRD, minimal residual disease; NS, non-significant; PTCy, post-transplant cyclophosphamide.

In multivariate analysis (**Table 7**), male patients having female donors had lower CD4^+^ T-cell counts (OR 0.2, 95% CI 0.04-0.93, *P*=0.04). As in univariate analysis, patients transplanted from a matched sibling donor had higher CD8^+^ and NKT cell counts (OR 6.23, 95% CI 1.64-23.73, *P*=0.007; OR 7.7, 95% CI 1.99-29.6, *P*=0.003). The association between EBMT risk score and NKT cells was also preserved (OR 0.13, 95% CI 0.02-0.73, *P*=0.02). However, when adding early CMV reactivation to the multivariate model, the association between CMV serostatus (+/+) and CD8^+^ T-cell count was lost, but the association with CD4^+^ T-cells remained (OR 0.13, 95% CI 0.04-0.49, *P*=0.03). We did not perform multivariate analysis for predicting B-cell and NK cell count since no variable proved statistically significant in univariate analysis.

**Table 7:** Multivariate regression analysis on immune reconstitution.

	CD4 IR			CD8 IR			NKT IR		
	*P*-value	OR	95% CI	*P*-value	OR	95% CI	*P*-value	OR	95% CI
Age (>median)	0.13	1.04	0.98-1.1	0.57	0.99	0.94-1.03	0.052	1.06	0.99-1.14
EBMT score (>2)	0.09	0.26	0.05-1.22	0.45	1.68	0.43-6.56	0.02^d^	**0.13**	**0.02-0.73**
Donor-patient sex	**0.04** ^a^	**0.2**	**0.04-0.93**	0.13	0.28	0.06-1.42	0.25	0.37	0.07-1.97
CMV serostatus	0.03^b^	**0.13**	**0.04-0.49**	0.35	1.69	0.56-5.1	0.57	0.69	0.2-2.4
Donor type	0.67	1.31	0.37-4.66	0.007^c^	**6.23**	**1.64-23.73**	0.003^c^	**7.7**	**1.99-29.6**
CMV reactivation	0.15	0.4	0.12-1.39	0.42	1.58	0.6-5.1	0.2	2.3	0.65-8.26

P values less than 0.05 are bolded.

a Lower counts in male patients with female donors vs others.

b Lower counts in CMV seropositive patients with a seronegative donor vs others.

c Higher counts in patients with a matched related donor vs others.

d Higher counts in patients with EBMT risk score less than 3.

EBMT score, European Society for Blood and Marrow Transplantation risk score;

## 4 Discussion

In this study, we aimed to analyze the correlation of lymphocyte subset reconstitution after T-cell depleted PBSCT with different clinical outcomes. We further tried to minimize the effects of common early post-transplantation complications that are known to influence both IR and transplant outcomes, such as acute GvHD, PGF, and GF, by excluding patients experiencing any of these events before lymphocyte subset measurement. In our carefully selected population, we demonstrated that NK cell counts above 178/µL on day 90 led to better transplant outcomes by improving OS and reducing NRM. Even though our population comprised of 70 patients, the results are significant because of the study design that selected a cohort homogenous for many variables previously shown to influence IR. All of the patients received *in vivo* TCD, and vast majority received ATG for this indication (66/70, 94%). Usage of TCD, as well as its modality (*in vivo* or *ex vivo*) represents one of the, if not the strongest predictor for immune reconstitution kinetics (12, 29, 38). The modality of GvHD prophylaxis also has an influence on IR (7) and all of our patients treated with ATG received the same GvHD prophylaxis with cyclosporine A and methotrexate. Faster IR is achieved when using peripheral blood as a stem cell source (4, 39), a modality used in all of our patients. It is also worth mentioning that all of the study patients were transplanted in the previous six years. In summary, by having a GvHD-free population receiving a homogenous stem cell source, TCD technique, and GvHD prophylaxis regimen, we minimized the influence of these variables on immune reconstitution kinetics and transplant outcomes. Since peripheral blood is the most common stem cell source and ATG still the most common instrument for TCD, we obtained results that could be generalized to a large population of transplant recipients.

High NK cell values were associated with reduced NRM, which is in line with the previous studies (15–17, 19, 20). Buhlmann et al. reported reduced rates of NRM in patients having high NK cell counts three months after transplantation, while using a pre-defined cut-off of >150 cells/µL (15). This effect was also maintained at six months, and it was comparable in magnitude with that of carrying >200 CD4^+^ cells/µL. We did not test the latter association, due to a very low number of patients achieving these counts (5/70, 7%), which can be attributed to the fact that the mentioned study evaluated T-replete grafts, in contrast to us. However, in our study, patients with CD4 values above the median of 56/µL had a trend toward lower rates of NRM (HR 0.37, 95% CI 0.12-1.15, *P*=0.08), which is similar to previous results demonstrating lower NRM when using a cut-off for CD4 IR of 50/µL (9, 25, 28–30). We could not replicate the association of high CD4 counts to improved OS, nor did we find a protective effect of high B-cell counts, which was implicated in one of the most recent studies (30). Since B-lymphocyte reconstitution might take up to five years (12), assessing its impact as early as three months after transplantation may be too soon to observe any meaningful benefit. Notably, Troullioud et al. demonstrated this protective effect in a pediatric cohort (median age 12.1), potentially due to a faster B-cell reconstitution in pediatric patients compared to adults (40) Similarly to our design, Savani et al. (16) used their median cut-off and demonstrated lower NRM and better OS in patients with high NK cell counts at day 30 (>150/µL). Our study, in addition to data from the literature, suggests that high NK cell counts might protect primarily from infectious complications, particularly of viral etiology (41). In line with this, all of our patients succumbing to infectious complications had low NK cell counts, although the number of deaths attributed directly to infection was too low to analyze as an end-point separately. Nevertheless, given that half of the infection-related deaths were attributed to CMV, it is noteworthy that the two groups showed similar rates of late CMV reactivation (40% in the NK IR group and 38% in the no NK IR group, *P*=1). This observation suggests a potential protective role of NK cells in mitigating CMV-related complications, including mortality. On the other hand, the mortality rate caused by GvHD complications was the same in both groups. Minculescu et al. found a significant correlation between NK cell counts on day 30 and acute GvHD grades II to IV, but the correlation disappeared when excluding patients diagnosed before day 30, implicating that low NK cell numbers in patients with acute GvHD is a result of treatment rather than a protective effect of NK cells in the pathogenesis of GvHD (17). In our study, patients with high NK cell counts showed a trend towards lower rates of sGF/PGF (22% vs 5.7%) with a borderline statistical significance (HR 0.23, 95% CI 0.05-1.02, *P*=0.053), an association that was previously reported (19). We could not demonstrate improved EFS in patients with high NK cell counts, which was due to the unexpectedly high rates of relapse in this group of patients. Improved OS was entirely mediated by lower NRM, as was the case in some of the previous studies (15, 17). Kim et al. showed strikingly similar results to ours, as high median NK cell counts on day 90 (>181.9/µL) predicted better OS and lower NRM, with no difference in PFS, while analyzing the same number of patients as us (20).

As mentioned, we did not replicate the previously reported association of NK IR and reduced relapse rates early after transplantation (16, 19). A recent study evaluating patients in the PTCy setting also demonstrated better OS with reduced relapse rates in patients with NK cell counts above the median (50.5/µL) at day 28 (21). In our population, there was a trend towards higher relapse rates in the group with NK IR. Given the previous reports stating that NK cell alloreactivity only affected relapse risk in myeloid leukemias (16), we performed a subgroup analysis in patients with AML, albeit with no statistically significant results. Association between high day 60 NK cell counts and improved relapse-free survival (RFS) was also previously reported, but only in patients receiving reduced intensity conditioning (18). We additionally performed this survival analysis, but, expectedly, found no difference in RFS between the groups (*P*=0.48, data not shown). In contrast to these studies, others found no correlation between high NK cell counts and relapse rates; or observed an association only when NK cell counts were measured 12 months post-transplantation (15, 17). It is worth noting that the strong association between early NK IR and reduced relapse rates was clearly demonstrated in *ex vivo* TCD studies, while studies done in T-replete grafts failed to replicate these findings. Furthermore, in the vast majority of cases, the impact on relapse was observed only when NK cell counts were measured one month after transplantation. In summary, neither study reported a protective effect of high NK cell counts on disease relapse when measured three months after transplantation, suggesting that earlier reconstitution is needed to achieve this effect.

We assessed a broad range of patient, disease, and transplant-related factors as potential predictors for IR in different lymphocyte subsets, aiming to identify possible confounding variables that could influence transplantation outcomes. Interestingly, no factor showed a statistically significant correlation with NK IR, further supporting the notion that NK IR was an independent predictor of improved outcomes in our population. Patients receiving a transplant from MSD had higher counts of CD8^+^ T-lymphocytes and NKT cells, which was not surprising given the lower dose of ATG used in this indication, although it is interesting that ATG dose did not affect CD4^+^ T-cell counts, contrary to the previous studies performed with Thymoglobulin^®^ (26). In univariate analysis, CMV seropositive patients with seropositive donors exhibited higher CD8^+^ T-cell counts. However, this association disappeared when CMV reactivation was included in a multivariate model, suggesting confounding, as the relationship between CD8+ T-cell counts and CMV reactivation has already been well-described (4, 41). Interestingly, lower CD4^+^ T-cell counts were observed in male recipients with female donors as well as in CMV seropositive patients with seronegative donors—all of which were previously linked to poorer transplant outcomes (42, 43).

Given the existing evidence mainly highlighting the importance of CD4^+^ T-cell and NK cell reconstitution in transplant outcomes, we focused our analysis on day 90 post-transplant, primarily because earlier CD4^+^ T-cell assessment would likely be uninformative in our population, which received *in vivo* T-cell depleted grafts, known to delay CD4^+^ T-cell recovery. However, knowing the rapid recovery kinetics of NK cells and their relevance in our findings, we additionally analyzed IR data on day 30 post-transplant, again defining IR of each lymphocyte subset as values above the median. The impact of day 30 IR on transplant outcomes is shown in **Supplementary Figures S1**-**S3**. Contrary to previous findings, we did not replicate a protective effect of high NK cell counts at day 30. It is worth noting that our median value was significantly lower to the median threshold of 150/µL reported by Savani et al. (16). This discrepancy may reflect key differences between the cohorts, primarily the use of Cyclosporine A without methotrexate for GvHD prophylaxis. Additional differences include the use of *in vitro* rather than *in vivo* T-cell depletion, and uniform use of matched sibling donors and total body irradiation-based conditioning regimens in their study. A specific design of our study, limited to patients not experiencing major complications in the first three months after transplantation, could further contribute to differences in results.

When designing the study, we faced with the dilemma of whether to use the predetermined IR cut-offs found in the literature. However, these are not yet standardized, resulting in a wide range of values used. For example, Kim et al. used cut-offs of 200/µL at three months for CD4^+^ T-cells by extrapolating data of infection risk from HIV literature. The cut-off for NK IR was set at 100/µL (3). On the other hand, Van Roessel et al. used a familiar cut-off of 50/µL for CD4^+^ T-cells, but thresholds defined for NK IR was 200/µL (29). Buhlmann et al. set a cut-off for NK IR at 150 cells/µL, whereas cut-offs for CD4^+^, CD8^+^ T-cells and B-cells were set at 200 cells/µL (15). Other studies used their own median values for defining the threshold for IR (6, 8, 10, 16). This goes to show that assessing data in this field is still not standardized, and this issue will have to be addressed in the future in order to get more consistent and coherent results across different studies. In the absence of an international consensus on predefined cut-off values, and based on our validation approach using Cox-restricted cubic spline plot analysis, which supported the use of median values as appropriate and meaningful cut-offs for predicting overall survival in our cohort, we based our analysis on the median values.

To this date, assessing IR isn’t the standard of care in all transplant centers. In a recent EBMT survey with participation of 76 centers, 82% of centers stated to test T cell subset counts by flow-cytometry, 53% test B cell counts, and only 39% test NK cell counts, with no data regarding the frequency of testing (44). Given that more than half of the centers still do not test NK cell counts after transplantation, this implicates a fairly unexploited potential of this relatively simple analysis. There is still no consensus on the optimal frequency and schedule for monitoring IR after transplantation, and whether this data can be used in clinical decision-making, again pointing out the fact that this is still a relatively unutilized area, which is yet not surprising given the complex interplay of events influencing it.

The retrospective nature of this analysis is a limitation and the data we obtained needs validation in a larger cohort. Although the study is retrospective, several factors contributed to a minimal extent of data loss: The data was prospectively entered into our institutional database, and all of our patients were transplanted recently-in the last seven years. Unfortunately, we did have eligible patients missing IR data on day 90 that could not be added to the analysis, but with a low drop-out rate (8.5%). Even though our study ultimately included 70 patients, the population was very uniform, as previously described, and data validity is further favored by the single-institution data retrieval. A further limitation is the fact that numerical assessment of IR is a simple biomarker, and functional assessment, if available, should be used in combination to obtain the most comprehensive assessment of the patients. The lack of standardizing flow cytometry-based methods remains an important barrier to reproducibility between studies, mainly due to differences in antibody clones, gating strategies and instrument configurations. To enhance reproducibility, we used a standard methodology for the detection and quantification of lymphocyte subsets, and we described our methodology in detail. In conclusion, we demonstrated that high NK cell counts at day 90 correlated with reduced NRM and improved OS in adult patients without prior major post-transplant complications receiving *in vivo* T-cell-depleted peripheral blood stem cell grafts. The results suggest a potential role for adoptive NK cell therapy, and a recent meta-analysis addressed this idea, while also demonstrating the impact of a higher graft NK cell content on improved transplant outcomes (22). These findings underscore the importance of NK cells in determining the success of allogeneic transplantation and imply that regular monitoring should serve as a cost-effective tool for decision-making and patient risk stratification.

## Data Availability

The raw data supporting the conclusions of this article will be made available by the authors, without undue reservation.

## Supplementary material

Supplementary Figure 1 Kaplan-Meier curves for overall survival (OS) **(A-E)** and event-free survival (EFS) **(F-J)** according to lymphocyte subpopulation counts on day 30 after transplantation. CD4-CD4^+^ T-cell, CD8-CD8^+^ T-cell, CD19-B-cell, IR-immune reconstitution, NK-NK cell, NKT-NKT cell.

Supplementary Figure 2 Cumulative incidence curves for non-relapse mortality (NRM) **(A-E)** and relapse incidence (RI) **(F-J)** according to lymphocyte subpopulation counts on day 30 after transplantation. CD4-CD4^+^ T-cell, CD8-CD8^+^ T-cell, CD19-B-cell, IR-immune reconstitution, NK-NK cell, NKT-NKT cell.

Supplementary Figure 3 Cumulative incidence curves for secondary graft failure/poor graft function (sGF/PGF) **(A-E)** and acute graft-versus-host-disease grade II-IV (aGvHD gr II-IV) **(F-J)** according to lymphocyte subpopulation counts on day 30 after transplantation. CD4-CD4^+^ T-cell, CD8-CD8^+^ T-cell, CD19-B-cell, IR-immune reconstitution, NK-NK cell, NKT-NKT cell.
