# Supplementary figures and images for: High NK cell counts at day 90 predict improved survival in event-free patients after T-cell depleted allogeneic stem cell transplantation
Source: Front Immunol. 2025 Jun 18;16:1577924. doi: 10.3389/fimmu.2025.1577924 (PMC12213383; doi:10.3389/fimmu.2025.1577924)

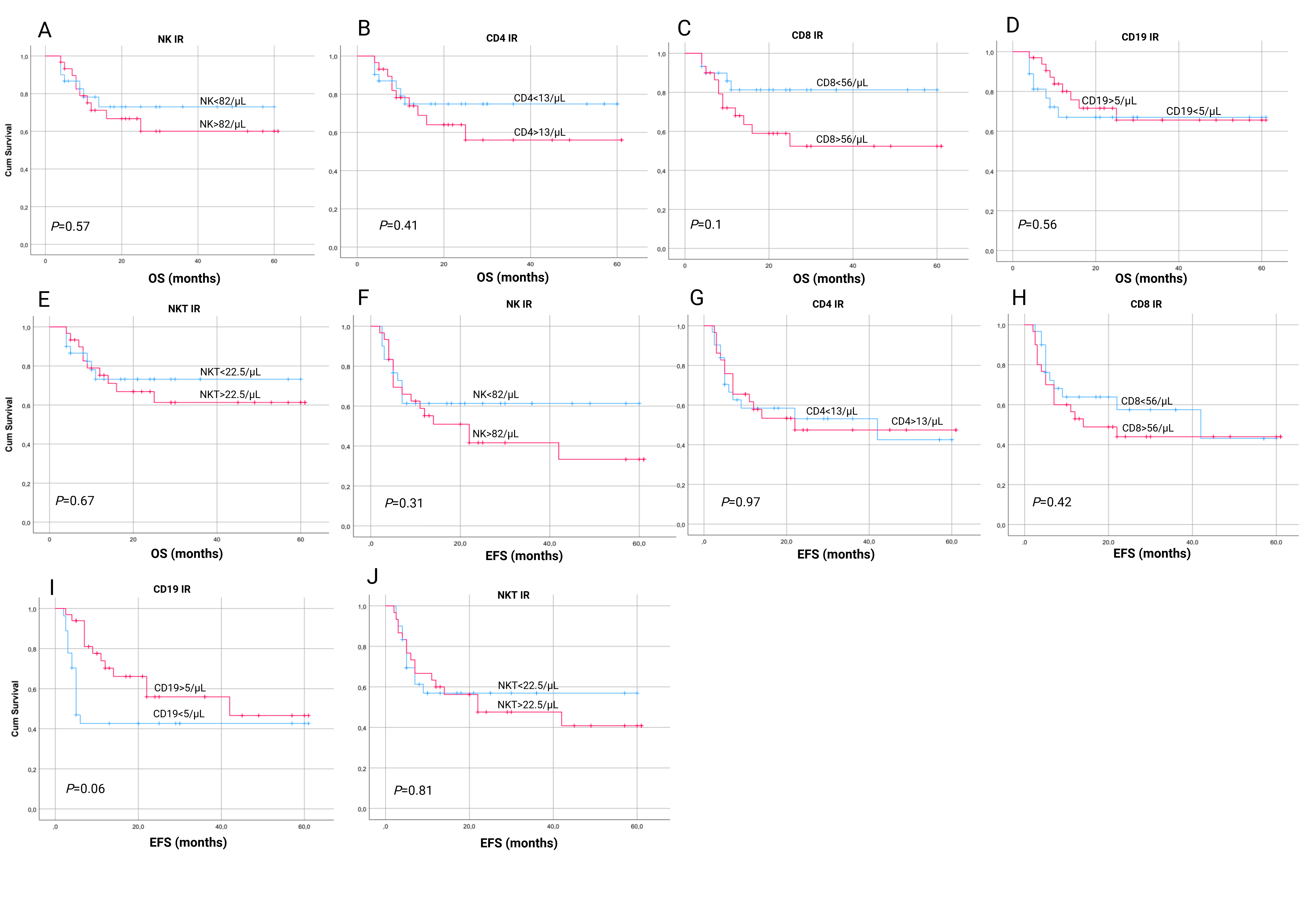

Supplement: Supplementary Figure 1 — Kaplan-Meier curves for overall survival (OS) (A-E) and event-free survival (EFS) (F-J) according to lymphocyte subpopulation counts on day 30 after transplantation. CD4-CD4+ T-cell, CD8-CD8+ T-cell, CD19-B-cell, IR-immune reconstitution, NK-NK cell, NKT-NKT cell. [file Image1.jpeg]

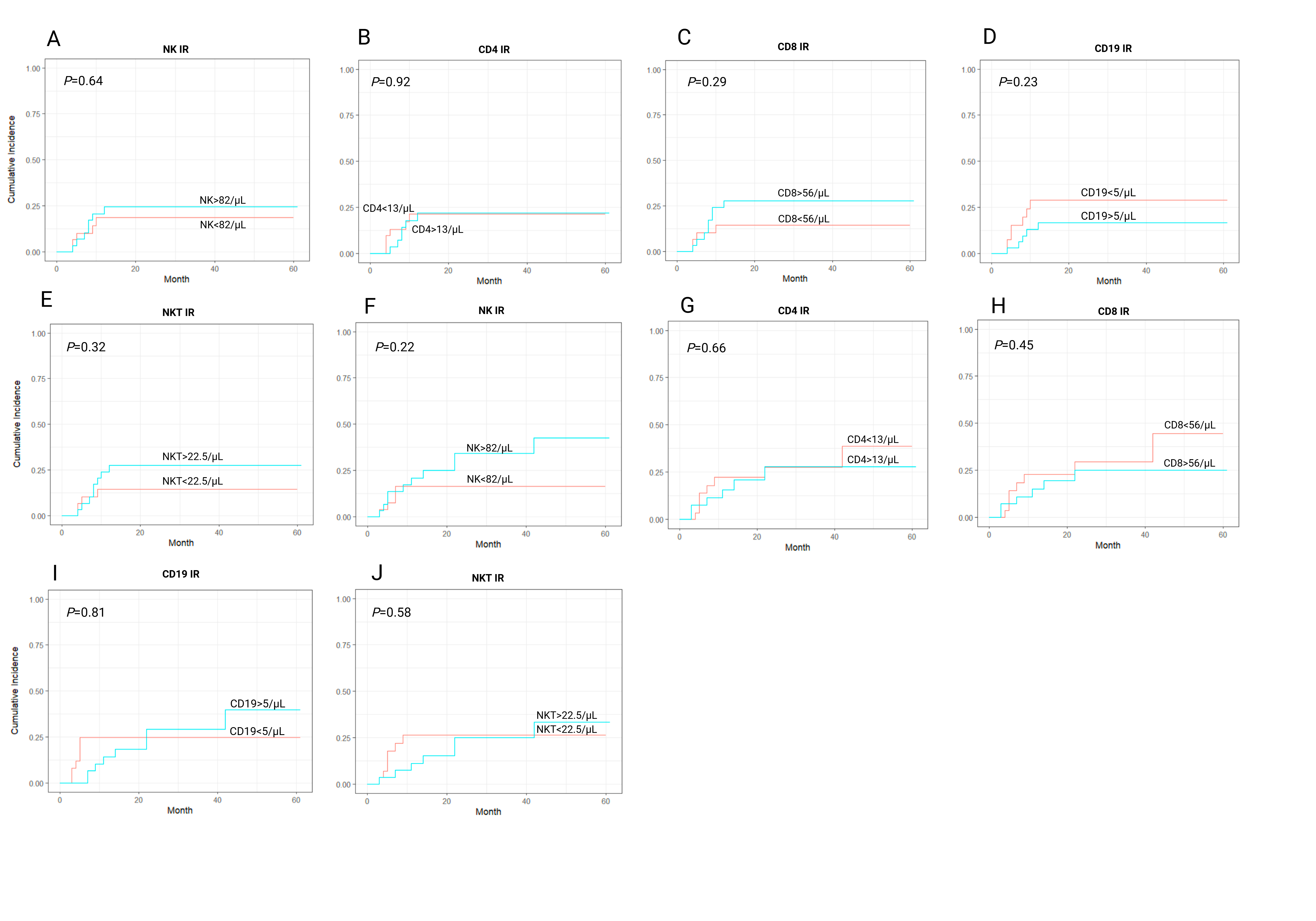

Supplement: Supplementary Figure 2 — Cumulative incidence curves for non-relapse mortality (NRM) (A-E) and relapse incidence (RI) (F-J) according to lymphocyte subpopulation counts on day 30 after transplantation. CD4-CD4+ T-cell, CD8-CD8+ T-cell, CD19-B-cell, IR-immune reconstitution, NK-NK cell, NKT-NKT cell. [file Image2.jpeg]

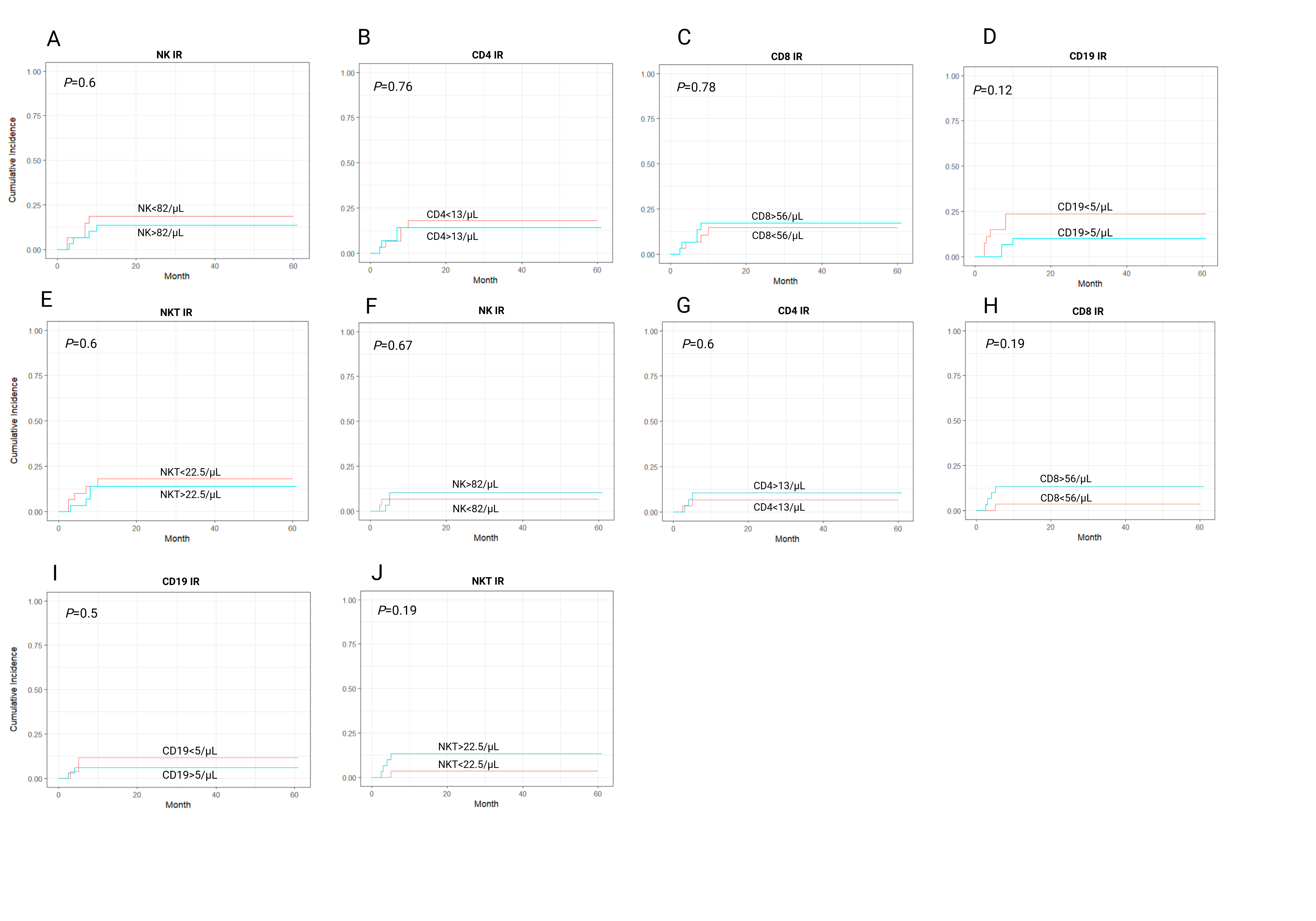

Supplement: Supplementary Figure 3 — Cumulative incidence curves for secondary graft failure/poor graft function (sGF/PGF) (A-E) and acute graft-versus-host-disease grade II-IV (aGvHD gr II-IV) (F-J) according to lymphocyte subpopulation counts on day 30 after transplantation. CD4-CD4+ T-cell, CD8-CD8+ T-cell, CD19-B-cell, IR-immune reconstitution, NK-NK cell, NKT-NKT cell. [file Image3.jpeg]
